# Experimental Study on Improving Wear Resistance by Hardfacing of Rotary Drying Segments Used in the Asphalt Industry

**DOI:** 10.3390/ma19071331

**Published:** 2026-03-27

**Authors:** Andrei Burlacu, Marius Gabriel Petrescu, Eugen Laudacescu, Mihaela-Mădălina Călțaru, Andreea-Mioara Dumitru, Marius Bădicioiu, Cristina Sescu-Gal

**Affiliations:** 1 Mechanical Engineering Department, Petroleum-Gas University of Ploiești, 100680 Ploiesti, Romania; andrei-ion@upg-ploiesti.ro (A.B.); leugen@upg-ploiesti.ro (E.L.); andreea.dumitru@upg-ploiesti.ro (A.-M.D.); mbadicioiu@upg-ploiesti.ro (M.B.); 2 Department of Construction Equipment and Advanced Technologies, Technical University of Civil Engineering Bucharest, 020396 Bucharest, Romania

**Keywords:** rotary dryer flights, hardfacing, wear-resistant materials, gas metal arc welding (GMAW), shielded metal arc welding (SMAW)

## Abstract

The asphalt industry, essential for the global transport infrastructure, requires substantial investments to increase the durability of production facilities. The quality of asphalt depends, essentially, on the degree of drying of mineral aggregates. Therefore, the rotary dryer is of major importance for ensuring the quality of asphalt. The rotary dryer flights are subjected to an erosive-abrasive wear process during operation, generated by the impact of abrasive aggregates. These phenomena lead to severe degradation of the flights. Experimental research, carried out by the authors, on-site, aimed at identifying solutions to improve the wear behavior of the flights, by hardfacing with four wear-resistant materials (FLUXOFIL 51, FLUXOFIL 56, SAFER R 400, SAFER R 600), using the GMAW and SMAW processes. The results revealed a decrease in the wear rate and a flattening effect of the wear curve along the profile of the flight. The research targeted the upper rear surface of the flights, which is predominantly affected by erosive-abrasive wear phenomena. The resistance to abrasive wear of the flights was improved by hardfacing with FLUXOFIL 51 wear-resistant tubular wire, resulting in the lowest wear rate, especially between the areas marked 14–26, which are the areas most affected during operation.

## 1. Introduction

**Figure 1:**
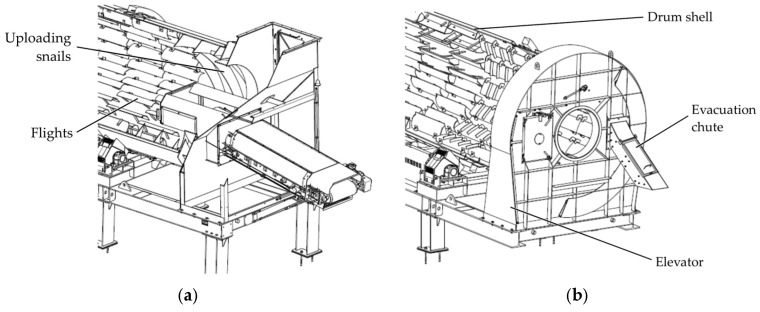
Schematic representation of the Benninghoven ECO 2000 rotary dryer divided into modules: (**a**) uploading section view; (**b**) downloading section view.

The industry of asphalt plays an important role and has an impact on the global economy, environmental protection, and people’s everyday life, especially due to population growth, rapid urbanization, and the necessity to improve and extend the global transportation infrastructure (motorways, highways, roads) in order to support economic activities and sustain connectivity. The total length of paved highways and roads exceeds 14 million km and continues to grow. In 2007, according to European Asphalt Pavement Association (EAPA), 5.2 million km were paved in Europe, 4.0 million km in the United States, 1.5 million km in China, 2.5 million km in the rest of Asia, 415,000 km in Canada, 344,000 km in Central and South America, 178,000 km in Mexico and, 64,000 km in Australia and New Zealand [1]. According to the market research reports, the global asphalt market is projected to reach 225.9 million tons by 2030, up from 158.5 million tons in 2024 [2], and is expected to increase from 563.3 million USD in 2025 to 676 million USD in 2030 [3]. In order to meet the growing market demands, as well as the legislative requirements and the quality standards, the companies continuously invest in research to improve, develop, and use different materials, technologies, and equipment in asphalt mixing plants. The asphalt mixing plants are the key components in the asphalt production process because they receive mineral aggregates (such as sand, crushed stone, gravel, recycled materials) and transform them into the final asphalt product by drying, heating, and mixing with the asphalt binder. The quality and characteristics of aggregates are essential to ensure high-quality asphalt pavement with strength and durability. In order to avoid premature pavement failure, it is very important to remove any moisture from the aggregates, as it can reduce adhesion to the asphalt binder and lead to the formation of weak spots [4]. Based on this, one of the most important components used in the asphalt production line is the direct rotary dryer that is responsible for removing moisture from the aggregates and heating them to temperatures typically between 135 and 200 degrees Celsius before the mixing process with the binder [4,5]. The rotary dryer is a steel cylindrical drum that rotates at different speeds and is slightly horizontally inclined (0–5 degrees) to ensure the longitudinal movement of the particles during the process (Figure 1). The drum’s interior features replaceable longitudinal blades of different sizes and shapes distributed around the entire circumference and along the full length of the cylinder, called flights, which lift, disperse, and drop the aggregates through the hot gas stream produced by a burner [6].

**Figure 2:**
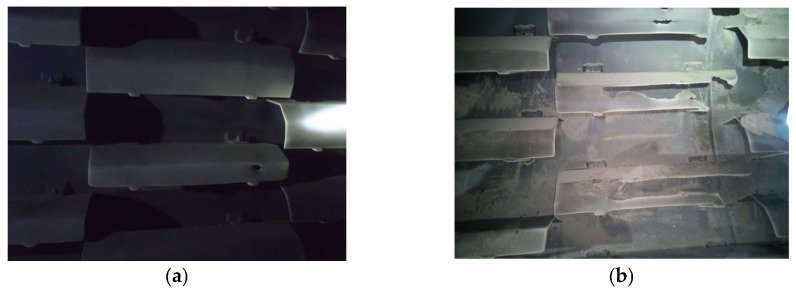
Rotary dryer flights: (**a**) new flights; (**b**) worn flights.

Worldwide specialists from different fields are interested in finding methods to improve the performance of rotary dryers, making the drying process more efficient in order to minimize pollution and energy consumption, in compliance with regulations concerning the manufacture of environmentally friendly and sustainable products [7,8]. According to specialists, the efficiency of the rotary dryer is influenced by drum dimensions, inclination, and flight configuration (shape and size), which affect particle dispersion inside the cylinder and heat transfer performance. Jeniffer Silviera et al. [9] investigated, by using the Discrete Element Method (DEM), the effects caused by angulations in three-segment flights on the particle dynamics, as well as the effect of the intercalated position of the flights on particle distribution along the drum length. According to Qiang Xie et al. [10], the rate of heat transfer inside the rotary dryer depends on the amount of heat transferred from the wall to the particles and on the heat transferred inside the particle flow, and any change in the lifters/flights (height, number, and shape) affects the total contact area significantly. It was observed that the total contact area increases by 44% when lifter height changes from 0 to 6 particle diameters and by 23% from 6 lifters to 15, and for the straight lifters is 11% higher than the arc lifters. Also, Mohamed A. Karali et al. [11] have shown that the maximum height of the falling material curtains is achieved when rotary drums operate at optimum loading. The study indicates that the number of flights and their length ratio have a greater influence on the filling capacity than the rotation speed (1–5 rpm) or the characteristics of the aggregates (sand, glass, steel particles with diameters of 0.2–2 mm). Other studies were focused on improving the efficiency and performance of the rotary dryer by increasing the service life of equipment and its components. During the exploitation work, the rotary dryer flights are subjected to a severe erosive-abrasive wear process, mainly due to abrasive particle impact and motion that produce structural changes in the material and cause superficial fatigue, leading to severe degradation of the flights [12,13]. Figure 2 shows images of the flights mounted inside a Benninghoven ECO 2000 rotary dryer, before and after the degradation process.

**Figure 3:**
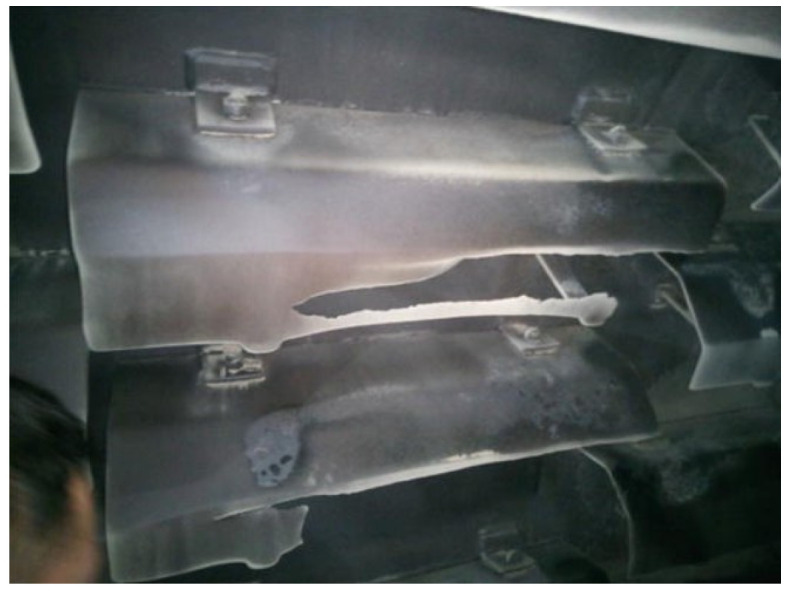
Non-uniform wear along the length of the flight profile [14].

As stated in a previous article [14], the wear of the flights is not uniform, being more intense on the upper rear surface of the flights (Figure 3).

As reported by Tudor A. et al. [15], the erosion mainly occurs in the form of scratches when the particle motion is parallel to the flight surface (small incidence angle), while micro-craters appear by tearing off material when the angle of incidence is around 90 degrees. At high incidence angles, particle velocity strongly influences the wear process generated by superficial fatigue of the surface material (at low speed), by plastic deformation or brittle fracture (at medium speed), or by plastic flow or even local melting of the target surface material (at high speed). In paper [16], the mathematical functions used to model the failure rates caused by wear of the flights are based on mass loss of material, reduction in the thickness of the part, and surface affected by wear. In order to improve the wear resistance of components, the hardfacing process—also known as hardbanding—is widely used by specialists in various industries for manufacturing or reconditioning processes. Through hardfacing, the surfaces of parts subjected to wear are covered with layers of wear-resistant materials using different welding technologies. Okechukwu, C. et al. [17] state that hardfacing is an economical method of extending the service life of worn components by reducing downtime, inventory, and maintenance costs, increasing plant availability and productivity, and providing a good number of service life extensions through timely repair. In paper [18], a hardbanding technology for reconditioning NC50 tool joints subjected to wear was established and validated, using a gas metal arc welding process (GMAW) with two wear-resistant wires, ARNCO 100XT and FLUXOFIL M58. Rajeev Ranjan [19] also noted that hardbanding performed using the GMAW process significantly improved the surface wear resistance of the part. The wear improvement of the part results from the optimal properties of the hardbanding layers (bead width, bead height, penetration, mechanical strength, wear resistance, corrosion resistance) obtained by the proper surface preparation and the adequate technological parameters (arc voltage, welding current, travel speed, stand-off distance, and welding gun angle). Yimiao Ning et al. [20] performed a consistent review of various hardfacing technologies and materials utilized in diverse applications to extend the durability of components subjected to abrasive and corrosive wear, reducing maintenance costs and improving operational efficiency. Based on the systematic review, the hardfacing techniques widely used are GMAW, shielded metal arc welding (SMAW), gas tungsten arc welding (GTAW), submerged arc welding (SAW), and plasma transfer arc welding (PTAW). The frequently used hardfacing materials are Fe-based, Ni-based, and Co-based alloys. The literature in the asphalt industry is focused mainly on improving the components’ efficiency based on their material, shape, dimensions, and working conditions. In contrast, it is extremely limited in terms of increasing durability by applying different technologies, especially hardfacing. This is the reason why the authors of the paper carried out experimental research in order to increase the wear resistance of rotary dryer flights through hardfacing of their active surfaces.

The main aim of the present study is to investigate, through experimental research, the possibility of decreasing the wear rate of rotary dryer flights used in the asphalt industry by hardfacing with four different wear-resistant materials (FLUXOFIL 51, FLUXOFIL 56, SAFER R 400, SAFER R 600), using the GMAW and SMAW processes.

## 2. Materials and Methods

### 2.1. Hardfacing of Rotary Dryer Flights

**Figure 4:**
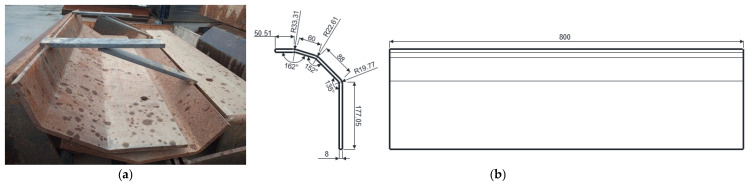
The original rotary dryer flights used in experimental research: (**a**) image; (**b**) schematic representation and dimensions.

The flights used in the experimental research are the original flights (Figure 4) usually used in the rotary dryer in the drying-dispersion zone, manufactured by rolling, with a thickness of 8 mm, made of steel grade S235JR [21], with the chemical composition presented in Table 1.

The chemical composition was determined using the Genius 5000 X-ray fluorescence spectrometer (Skyray Instruments, Dallas, TX, USA) through positive material identification (PMI).

The active (rear) surfaces of the flights, directly subjected to the impact of aggregate particles during exploitation work, were hardfaced with four different types of wear-resistant materials (Table 2) using the GMAW and SMAW welding processes, in accordance with the types of filler materials (wire or electrode) used. According to the manufacturer, all four wear-resistant materials used in this study are recommended for hardfacing parts subjected to wear and for the reconditioning process. The hardfaced layers were deposited along the length of the flight profile, where the wear is non-uniform, as demonstrated in practice. The hardfacing process was carried out by specialists at DUCTIL S.A. Buzau (Buzau, Romania), the manufacturer of the filler materials.

**Figure 5:**
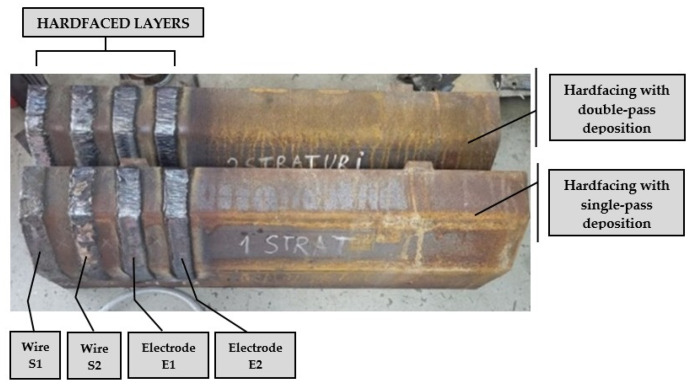
The hardfaced rotary dryer flights.

The rotary dryer flights were thoroughly cleaned prior to hardfacing by degreasing with an organic solvent (Safe Clean Eco, Tech Masters Trading SRL, Oradea, Romania), blowing with compressed air, rinsing with methyl-ethyl-ketone (MEK, CHIMREACTIVE SRL, Bucharest, Romania), and wiping with a solvent-resistant microfiber cloth. Four hardfaced layers were manually deposited on the surface of the rotary dryer flights, each layer corresponding to one of the four filler materials used, in either single-pass or double-pass deposition (Figure 5).

Table 3 presents the technological parameters and the identification codes used during the hardfacing process of the rotary dryer flights.

### 2.2. Operation of Hardfaced Rotary Dryer Flights on Site, Under Real Conditions

**Figure 6:**
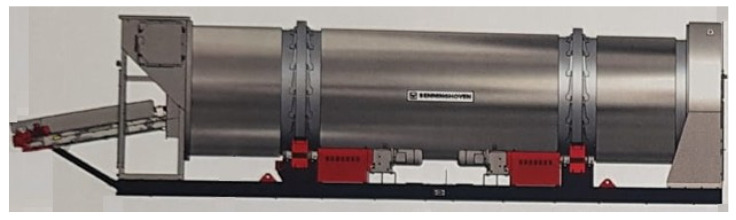
Configuration of BENNINGHOVEN rotary dryer [26].

The experimental research was performed on-site, in real conditions, on hardfaced flights mounted inside a Benninghoven ECO 2000 rotary dryer (BENNINGHOVEN GmbH & Co. KG, Wittlich, Germany) [26], having an internal diameter of 2200 mm, a wall thickness of 12 mm, and a drum length of 8000 mm (Figure 6).

**Figure 7:**
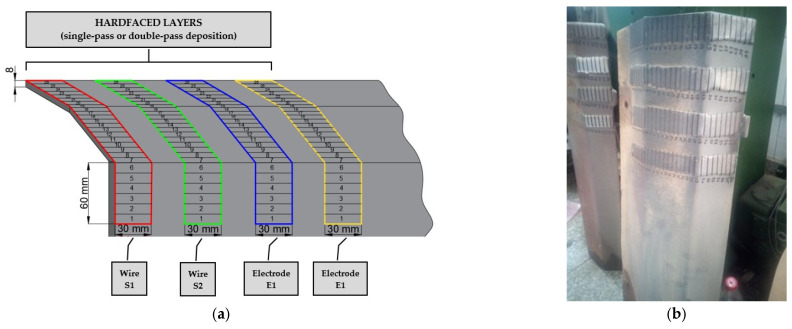
Arrangement of the marked grid for thickness and hardness measurements: (**a**) schematic representation; (**b**) hardfaced rotary dryer.

The rotary dryer is operated by STRABENBAU LOGISTIC S.R.L (Blejoi, Romania) and is manufactured by Benninghoven GmbH & Co KG (Wittlich, Germany). The rotary dryer operates with a capacity of 145–320 tons/h and a rotational speed of 7.5–10 rpm. Mineral aggregates with a grain size of 4–32 mm were used during the drying and heating processes. The hardfaced flights were prepared before being mounted in the rotary dryer. Thus, each hardfaced layer was marked along the entire length of the deposition to divide it into 26 equally wide zones, necessary for thickness and hardness measurements (Figure 7). The same grid was marked on the original flights (without deposition).

**Figure 8:**
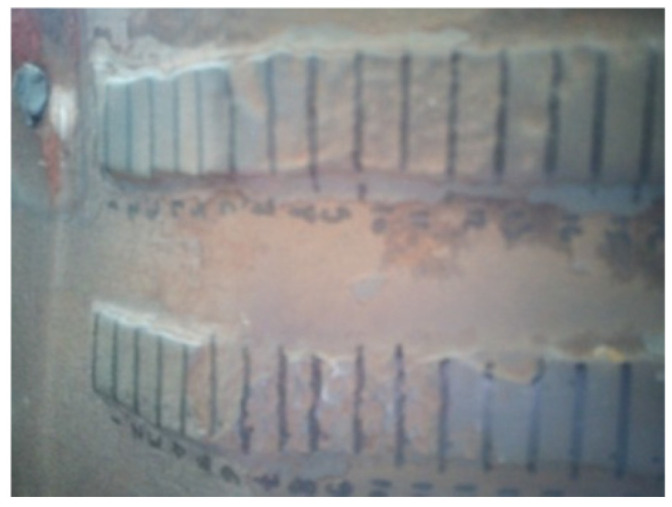
Marking of worn surfaces for thickness and hardness measurements.

The hardfaced flights, prepared according to the above specifications, were mounted in the rotary drying drum and operated for 1150 h in the presence of mineral aggregates. During the operating time, there were two interruptions of the equipment necessary for thickness measurement, after 300 h and 730 h. Thus, the flights were removed from the rotary dryer, washed with methyl-ethyl-ketone (MEK), wiped with a solvent-resistant microfiber cloth, and dried with hot air. After the cleaning process, the marked grid was reapplied (Figure 8).

## 3. Results and Discussion

### 3.1. Hardness Measurements of Hardfaced Flights

The hardness of the material is a very important characteristic with direct influence on its wear resistance. Vickers hardness measurements were performed by the authors in the 26 marked zones on both the original flights (without deposition) and the hardfaced layers of the rotary dryer flights, before and after the drying process. The hardness measurements were performed using the LEEB ISH-PHB-B INSIZE (INSIZE EUROPE S.L., Derio, Spain) portable equipment, and the average values of the 26 measurements are presented in Table 4.

By using the hardfacing process with wear-resistant materials, the hardness of the original rotary dryer flights (without deposition) made of steel grade S235JR (probe S) increases. In addition, hardfacing with double-pass deposition (probe F51-2, F56-2, E400-2, E600-2) results in higher hardness values than the single-pass deposition (probe F51-1, F56-1, E400-1, E600-1), but this aspect does not guarantee an improvement in the wear behavior of the flights. The hardfaced layer deposited with tubular wire FLUXOFIL 51 (probe F51), using the GMAW process, shows almost the same behavior as the electrode SAFER R 400 (probe E400), deposited with the SMAW process, in terms of hardness. The same behavior was observed for flights hardfaced with tubular wire FLUXOFIL 56 (probe F56) and electrode SAFER R 600 (probe E600), with similar results for the hardness values. The hardening degree, based on the percentage of hardness increase, of the rotary dryer flights after 1150 h of operation, does not provide relevant information regarding the improvement of wear performance through hardfacing.

### 3.2. Thickness Measurements of the Hardfaced Rotary Dryer

In order to evaluate the material loss of the flights due to the impact with mineral aggregates during the operation, the authors performed thickness measurements in the 26 marked zones (points of measurement) on flights (with and without deposition). The thickness measurements of the hardfaced flights were conducted at four different periods of time, thus: before mounting them in the rotary drum (initial state), during the drying operation (intermediate state), after 300 h and 730 h when the process was interrupted, and at the end of the process after 1150 h (final state). An Olympus 38DL PLUS (Olympus NDT, Inc., Waltham, MA, USA) ultrasonic thickness gauge was used for thickness measurements, equipped with an M112-SM transducer with a frequency of 10 MHz and a diameter of 6 mm. Table 5 presents the values of the thickness measurements carried out on the original flight without deposition (probe S), at one end of the hardfaced flights.

As expected, the rotary dryer flights are subjected to intensive wear during the drying process, caused by impact with mineral aggregates that lead to material loss observed by a significant decrease in flight thickness until 60% after 1150 h of operation.

Table 6 shows the results of thickness measurements performed on the hardfaced flights for each individual hardfaced layer deposited in a single pass using different wear-resistant materials. According to results presented in Table 6, the hardfaced flights have, in the initial state, a non-uniform thickness because the hardfaced layers were deposited manually during the welding process, and the length of the electric arc was not kept constant, with direct influence on the weld pool and the dimensional characteristics of the layers.

The thickness of the hardfaced flights decreases during the operation time due to the impact with mineral aggregates. During the operation, the thickness of the hardfaced flight in different marked zones decreased to below 8 mm, which corresponds to the thickness of the original flight (without deposition). This is due to the exfoliation of the hardfaced layers and the action of mineral aggregates directly on the base material of the flight. The exfoliation of the hardfaced layer can result from improper preparation of the flight surface before hardfacing or due to the high velocity impact of the mineral aggregates, combined with the particle mass and their random movement.

Table 7 presents the results of thickness measurements performed on the hardfaced flights for each individual hardfaced layer deposited with double-pass using different wear-resistant materials. The thickness of the hardfaced flights before being mounted in the rotary drum is non-uniform due to the manual welding process used for hardfacing with double pass deposition. During the drying operation of mineral aggregates, the thickness of the hardfaced flights decreased. Comparatively with single-pass deposition, the double-pass deposition brings an improvement in the thickness reduction below 8 mm in the case of hardfaced flights with tubular wires FLUXOFIL 51 (probe F51-2) and FLUXOFIL 56 (probe F56-2). The hardfaced layers with double-pass deposition on the rotary dryer flights do not improve the thickness reduction below 8 mm when the electrodes SAFER R 400 (probe E400-2) and SAFER R 600 (probe E600-2) are used as filler material for the hardfacing process.

### 3.3. Wear Rate and Wear Distribution Along the Flight Profile

**Figure 9:**
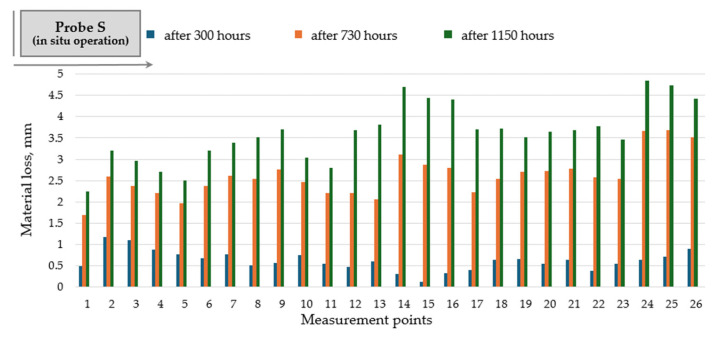
Wear distribution along the flight profile for the original rotary dryer flight made of steel grade S235JR (probe S).

The evolution of material loss during the operation of the original flight confirms the development of non-uniform wear distribution along the flight profile, with the most affected areas corresponding to the marked zones 14–26 (Figure 9).

During the drying operation, the surface of the flight is damaged due to abrasive wear because the action of mineral aggregates. The amount of material loss from a surface is a measure of the degree of abrasive wear. Thus, the wear distribution along the flight profile is in direct relationship with the thickness reduction in the components. Material loss is reported to the initial thickness of the rotary dryer flight.

**Figure 10:**
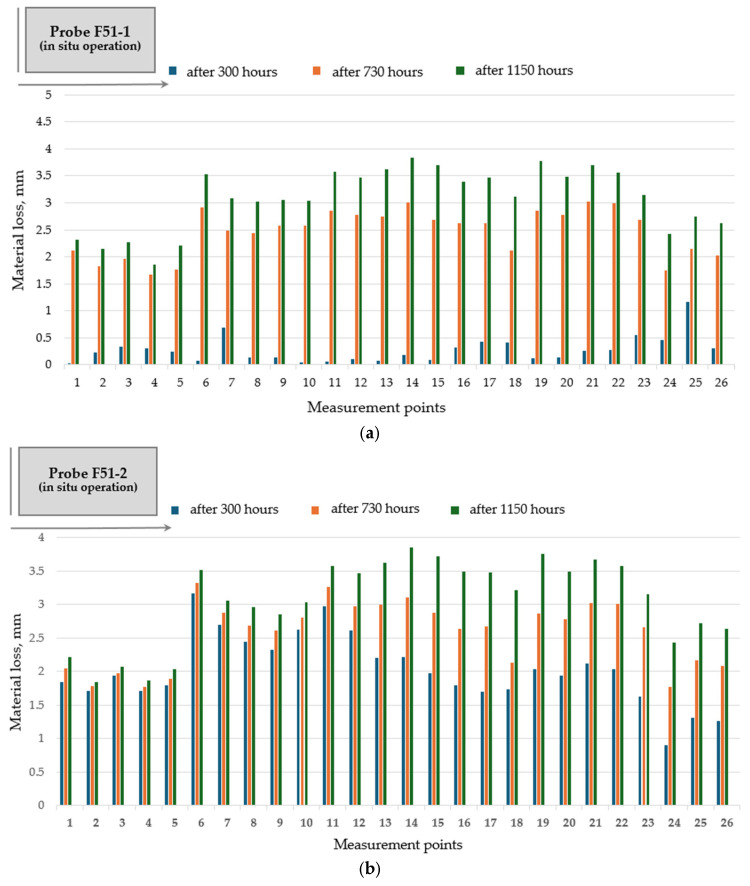
Wear distribution along the flight profile for hardfaced flight (probe F51): (**a**) single-pass deposition of tubular wire FLUXOFIL 51 (probe F51-1); (**b**) double-pass deposition of tubular wire FLUXOFIL 51 (probe F51-2).

**Figure 11:**
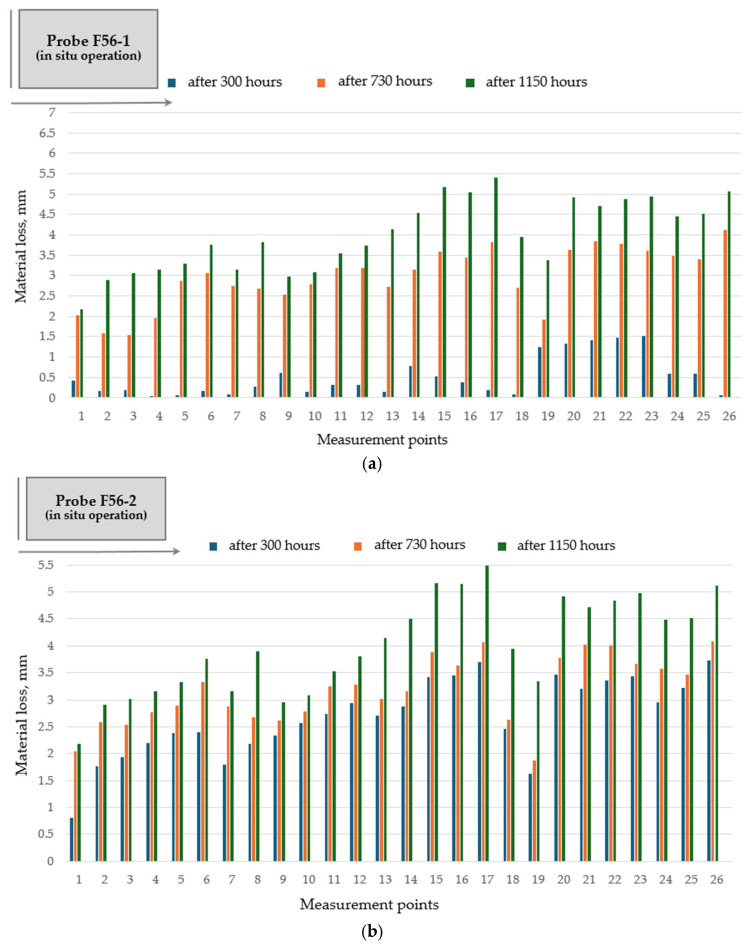
Wear distribution along the flight profile for hardfaced flight (probe F56): (**a**) single-pass deposition of tubular wire FLUXOFIL 56 (probe F56-1); (**b**) double-pass deposition of tubular wire FLUXOFIL 56 (probe F56-2).

Hardfacing of the rotary dryer flights with basic flux cored tubular wires FLUXOFIL 51 and FLUXOFIL 56 has a favorable impact on wear distribution along the flight profile (Figure 10 and Figure 11), especially in the case of FLUXOFIL 51. The use of FLUXOFIL 51 wire for hardfacing of the flight surface subjected to wear indicates a flattening (uniforming) effect on the pronounced wear developed between marked zones 14–26, which are the most affected areas during operation. Hardfacing with double-pass deposition does not lead to important results regarding the wear resistance improvement, and therefore, its application to the rotary dryer flights is unjustified.

**Figure 12:**
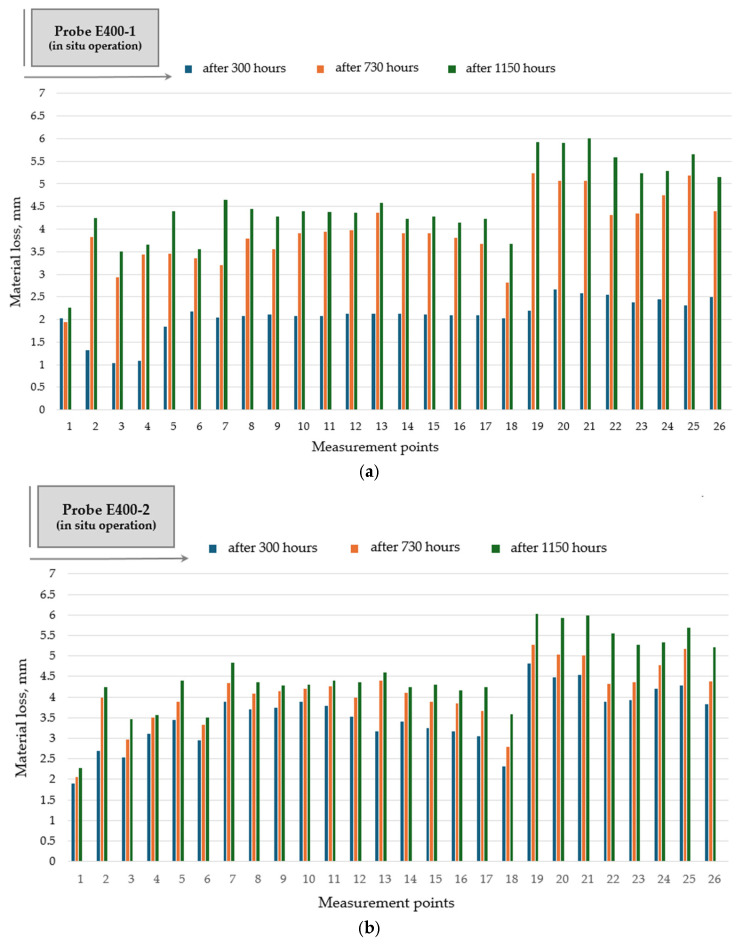
Wear distribution along the flight profile for hardfaced flight (probe E400): (**a**) single-pass deposition of electrode SAFER R 400 (probe E400-1); (**b**) double-pass deposition of electrode SAFER R 400 (probe E400-2).

**Figure 13:**
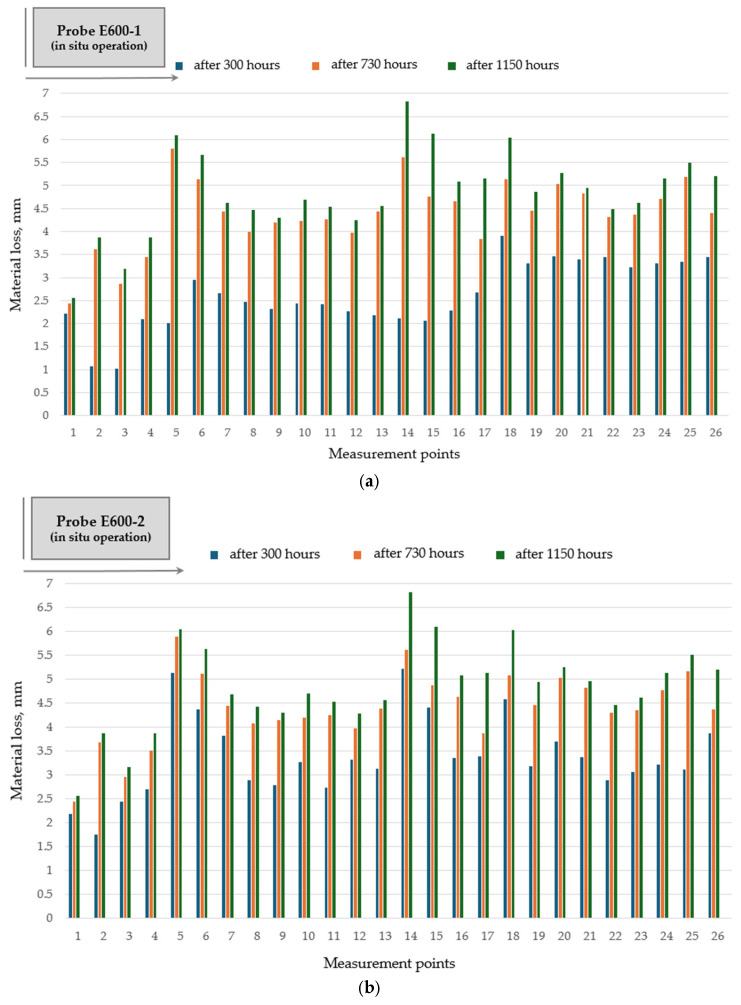
Wear distribution along the flight profile for hardfaced flight (probe E600): (**a**) single-pass deposition of electrode SAFER R 600 (probe E600-1); (**b**) double-pass deposition of electrode SAFER R 600 (probe E600-2).

Hardfacing of the rotary dryer flights with electrodes SAFER R 400 and SAFER R 600 does not bring any favorable effect to abrasive wear reduction and to wear distribution profile along the flight (Figure 12 and Figure 13).

The hardfaced flights with these wear-resistant materials, SAFER R 400 and SAF-ER R 600, reveal less favorable wear behavior than the original flights (without deposition). A possible explanation can be attributed to improper welding conditions, such as an insufficiently cleaned surface of the rotary dryer flight before welding, manual welding on complex geometry of the flight surface affecting the stability of welding parameters (amperage, voltage), or insufficient protection of the molten weld due to the absence of shielding gas during the SMAW process.

This study also aimed to investigate the influence of the hardfacing process on the wear rate of the rotary dryer flights during operation.

**Figure 14:**
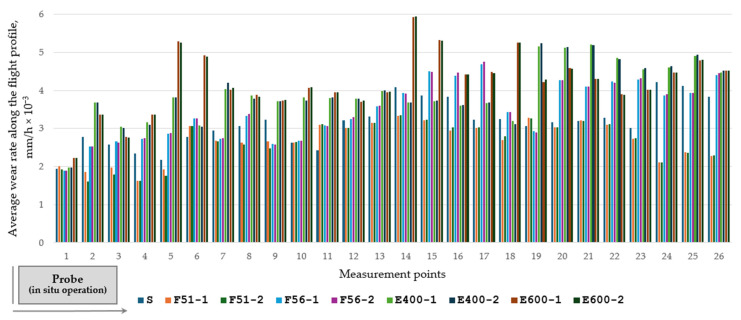
Average wear rate along the flight profile for original rotary dryer flight (steel grade S235JR without deposition) and hardfaced flights in single-pass or double-pass deposition with different wear-resistant materials (FLUXOFIL 51, FLUXOFIL 56, SAFER R 400, and SAFER R 600).

Based on the experimental investigations and results, the average wear rate along the flight profile was expressed as material loss (thickness reduction) per hour of operation (Figure 14).

Due to the random abrasive wear process produced by the impact of mineral aggregates on the rotary dryer flights, the wear rates are different from one flight to another when referring to the same marked zone on their surfaces.

The abrasive wear resistance of the rotary dryer flights is improved by hardfacing with wear-resistant tubular wire FLUXOFIL 51, resulting in the lowest wear rate, especially between marked zones 14–26, which are the most affected areas during operation. The wear rate of the hardfaced rotary dryer flights is not influenced by the number of deposition passes during the hardfacing process with wear-resistant materials, so double-pass application to the rotary dryer flights is unjustified.

## 4. Conclusions

The service life of the rotary dryer flights subjected to abrasive wear due to mineral aggregates impact during operation can be improved through the hardfacing process applied by welding with wear-resistant materials.

The results of experimental research performed on-site, under real conditions, on hardfaced flights mounted inside a Benninghoven ECO 2000 rotary dryer, revealed a decrease in wear rate along the flight profile. The average wear rate, estimated as the material loss (flight thickness reduction) per hour of operation, depends on the filler materials used for hardfacing and the welding technologies applied (surface preparation, welding parameters, manual or automatic welding). Due to the impact of mineral aggregates, combined with the particle mass and their random motion, the wear distribution profile is non-uniform along the flight profile, being more intense in the marked zones 14–26 (upper rear surface of the flights, as in the original flight without deposition).

Of the four wear-resistant materials (FLUXOFIL 51, FLUXOFIL 56, SAFER R 400, SAFER R 600) used for hardfacing of the rotary dryer flights, the basic flux core tubular wires FLUXOFIL 51 can be applied and are recommended for improving the flight durability during the drying operation. The results obtained in the frame of this research work on hardfaced flights with FLUXOFIL 51, in a single-pass deposition, showed:

- a flattening (uniforming) effect on the pronounced wear developed between marked zones 14–26, which are the most affected areas during operation;
- the lowest wear rate, especially between marked zones 14–26, which are the most affected areas during operation.

A double-pass deposition of the hardfaced layer is not recommended—did not demonstrate a significant effect of increasing wear resistance—having a similar influence to the rotary dryer flights’ performance obtained in a single-pass deposition, and is therefore unjustified.

The hardfacing procedure can be successfully used in the reconditioning operations of horizontal rotary dryer flights.

The deposition of hard materials by shielded manual arc welding (SMAW) leads to poorer results, mainly due to the lack of a protective environment during welding. From this point of view, welding in a protective gas environment (GMAW) proves to offer much superior results.

The authors have sought, in particular, solutions that can be applied in the repair shops of asphalt mixture manufacturing companies. It should be taken into account that, inside the rotary dryer, not all flights wear with the same intensity. Therefore, the solution proposed by the authors of the article tries to respond, in particular, to a user requirement related to preventive maintenance practices.

## Figures and Tables

**Table 1:** Chemical compositions of the steel grade S235JR [14].

Chemical Composition, %									
C	Si	Mn	P	S	Cr	Mo	Ni	Nb	V
0.1710	0.0320	1.370	0.0106	0.0032	0.0279	0.0017	0.0205	0.0004	0.0029

**Table 2:** Filler materials characteristics used for hardfacing process [22,23,24,25].

Filler Material Type	Chemical Composition, %				
	C	Mn	Si	Cr	Mo
FLUXOFIL 51 (basic flux core tubular wires, Ø 1.2 mm)	0.2	1.6	0.6	1.4	-
FLUXOFIL 56 (basic flux core tubular wires, Ø 1.6 mm)	0.4	1.7	0.6	6.0	0.7
SAFER R 400 (electrode, Ø 3.2 mm × 450 mm)	0.1	0.6	0.3	2.4	-
SAFER R 600 (electrode, Ø 3.2 mm × 450 mm)	0.6	1.1	1.0	2.8	-

**Table 3:** Hardfacing technological parameters and identification codes.

Filler Material	Identification Probe *	Number of Hardfaced Layers	Welding Parameter (Average Value)
Wire S1 (FLUXOFIL 51)	F51-1	1	Amperage: I_s_ = 160 A Voltage: U_a_ = 20.8 V Shielding gas: 100% CO_2_
	F51-2	2	
Wire S2 (FLUXOFIL 56)	F56-1	1	Amperage: I_s_ = 200 A Voltage: U_a_ = 20.6 V Shielding gas: 100% CO_2_
	F56-2	2	
Electrode E1 (SAFER R 400)	E400-1	1	Amperage: I_s_ = 85 A Voltage: U_a_ = 19.2 V
	E400-2	2	
Electrode E2 (SAFER R 600)	E600-1	1	Amperage: I_s_ = 110 A Voltage: U_a_ = 20.8 V
	E600-2	2	

* Identification probe S is used for original flights (without deposition) made by steel grade S235JR.

**Table 4:** Vickers hardness measurements of the hardfaced flights (average values of 26 measurements for each probe).

Identification Probe	Vickers Hardness of the Hardfaced Flights, HV		Hardness Increase, %
	Initial State (Before Being Mounted in the Rotary Dryer)	Final State (After 1150 h of Operation in the Rotary Dryer)	
S	149	187	13
F51-1	474	498	5
F56-1	679	689	1.5
E400-1	463	533	15
E600-1	670	714	6.5
F51-2	538	567	5.3
F56-2	679	731	7.6
E400-2	474	524	10.5
E600-2	715	729	2

**Table 5:** Thickness measurements of the flights without deposition (probe S).

Point of Measurement	Flight Thickness Without Deposition, mm			
	Initial State	After 300 h (Intermediate State)	After 730 h (Intermediate State)	After 1150 h (Final State)
1	8	7.52	6.32	5.76
2	8	6.83	5.41	4.8
3	8	6.91	5.63	5.04
4	8	7.12	5.79	5.3
5	8	7.24	6.03	5.5
6	8	7.32	5.63	4.8
7	8	7.23	5.38	4.61
8	8	7.50	5.47	4.48
9	8	7.43	5.24	4.29
10	8	7.25	5.54	4.97
11	8	7.45	5.8	5.21
12	8	7.53	5.8	4.31
13	8	7.41	5.95	4.19
14	8	7.69	4.88	3.30
15	8	7.88	5.12	3.55
16	8	7.67	5.21	3.59
17	8	7.61	5.78	4.29
18	8	7.37	5.46	4.27
19	8	7.34	5.3	4.48
20	8	7.45	5.27	4.36
21	8	7.37	5.22	4.32
22	8	7.63	5.42	4.22
23	8	7.45	5.46	4.54
24	8	7.36	4.34	3.15
25	8	7.29	4.32	3.27
26	8	7.11	4.49	3.58

**Table 6:** Thickness measurements of the hardfaced flights with single-pass deposition.

Point of Measurement	Flight Thickness with Single-Pass Deposition, mm *															
	Identification Probe															
	F51-1	F56-1	E400-1	E600-1	F51-1			F56-1			E400-1			E600-1		
	Initial State	Initial State	Initial State	Initial State	After 300 h	After 730 h	After 1150 h	After 300 h	After 730 h	After 1150 h	After 300 h	After 730 h	After 1150 h	After 300 h	After 730 h	After 1150 h
1	9.84	11.68	10.11	9.92	9.81	7.72	7.53	11.26	9.65	9.51	8.08	8.16	7.84	7.71	7.48	7.36
2	10.03	11.22	9.77	9.10	9.80	8.20	7.89	11.05	9.64	8.32	8.45	5.95	5.53	8.03	5.49	5.22
3	10.05	10.30	9.40	9.13	9.71	8.08	7.78	10.12	8.75	7.24	8.37	6.47	5.90	8.11	6.26	5.94
4	9.94	9.95	9.36	9.21	9.63	8.27	8.08	9.91	7.98	6.81	8.27	5.93	5.71	7.12	5.76	5.34
5	9.74	9.82	9.31	9.40	9.50	7.98	7.53	9.76	6.95	6.53	7.47	5.86	4.92	7.39	3.59	3.31
6	9.78	9.90	9.18	9.50	9.71	6.86	6.25	9.73	6.84	6.14	7.01	5.83	5.63	6.55	4.37	3.84
7	10.02	9.91	9.22	9.29	9.33	7.54	6.94	9.83	7.16	6.77	7.17	6.02	4.58	6.63	4.85	4.66
8	9.42	10.27	9.20	8.98	9.29	6.98	6.40	9.99	7.59	6.44	7.13	5.41	4.75	6.50	4.99	4.51
9	9.42	10.52	9.24	8.84	9.28	6.84	6.36	9.90	7.98	7.54	7.13	5.69	4.96	6.53	4.64	4.54
10	9.45	10.35	9.23	8.98	9.41	6.87	6.42	10.20	7.57	7.26	7.16	5.33	4.84	6.55	4.75	4.29
11	9.50	10.14	9.29	8.87	9.44	6.64	5.93	9.82	6.95	6.60	7.21	5.35	4.92	6.45	4.60	4.33
12	9.57	10.02	9.47	8.80	9.46	6.79	6.11	9.70	6.83	6.29	7.34	5.49	5.11	6.53	4.82	4.55
13	9.60	10.11	9.63	8.79	9.53	6.86	5.98	9.96	7.39	5.98	7.50	5.27	5.05	6.61	4.35	4.24
14	9.65	10.45	9.60	8.90	9.46	6.64	5.81	9.68	7.31	5.92	7.47	5.69	5.37	6.79	3.29	2.08
15	9.67	10.39	9.61	8.95	9.58	6.98	5.98	9.87	6.81	5.21	7.50	5.71	5.33	6.88	4.19	2.83
16	9.68	10.20	9.56	9.16	9.36	7.06	6.29	9.83	6.76	5.15	7.47	5.76	5.42	6.87	4.51	4.07
17	9.73	9.78	9.52	9.48	9.30	7.11	6.26	9.59	5.95	4.38	7.43	5.85	5.30	6.81	5.65	4.32
18	9.63	9.62	9.43	9.27	9.21	7.52	6.52	9.53	6.92	5.67	7.40	6.61	5.75	5.37	4.13	3.23
19	9.61	10.93	9.51	9.64	9.49	6.76	5.83	9.68	9.02	7.56	7.32	4.28	3.58	6.34	5.19	4.78
20	9.56	11.11	9.53	8.92	9.43	6.78	6.08	9.78	7.48	6.20	6.86	4.46	3.63	5.45	3.89	3.64
21	9.59	11.13	9.51	8.97	9.33	6.57	5.90	9.71	7.29	6.42	6.93	4.45	3.51	5.57	4.14	4.02
22	10.28	11.12	9.20	9.08	10.01	7.29	6.72	9.64	7.34	6.25	6.66	4.89	3.62	5.63	4.76	4.60
23	10.62	10.77	9.01	8.77	10.07	7.93	7.48	9.25	7.15	5.83	6.63	4.67	3.78	5.55	4.41	4.14
24	10.52	10.41	9.15	8.90	10.06	8.77	8.10	9.82	6.92	5.96	6.70	4.41	3.86	5.60	4.19	3.75
25	11.60	10.27	9.39	9.13	10.44	9.45	8.86	9.68	6.87	5.75	7.08	4.20	3.74	5.79	3.94	3.63
26	10.71	9.85	9.63	8.65	10.41	8.69	8.09	9.78	5.74	4.78	7.14	5.24	4.48	5.21	4.24	3.44

* The grey cells indicate the thickness of the hardfaced flights below 8 mm (corresponding to probe S, the original flight without deposition).

**Table 7:** Thickness measurements of the hardfaced flights with double-pass deposition.

Point of Measurement	Flight Thickness with Double-Pass Deposition, mm *															
	Identification Probe															
	F51-2	F56-2	E400-2	E600-2	F51-2			F56-2			E400-2			E600-2		
	Initial State	Initial State	Initial State	Initial State	After 300 h	After 730 h	After 1150 h	After 300 h	After 730 h	After 1150 h	After 300 h	After 730 h	After 1150 h	After 300 h	After 730 h	After 1150 h
1	13.30	14.30	10.77	9.39	11.46	11.25	11.09	13.49	12.25	12.12	8.87	8.72	8.50	7.20	6.94	6.83
2	13.11	15.07	12.16	11.34	11.4	11.33	11.27	13.30	12.49	12.17	9.46	8.18	7.93	9.59	7.66	7.46
3	13.10	14.66	11.83	11.05	11.16	11.13	11.03	12.73	12.13	11.64	9.29	8.86	8.36	8.61	8.08	7.88
4	13.09	14.16	11.63	10.94	11.38	11.32	11.23	11.96	11.39	11.01	8.52	8.12	8.06	8.23	7.43	7.07
5	13.12	13.41	11.64	11.09	11.33	11.23	11.09	11.03	10.52	10.09	8.19	7.75	7.25	5.96	5.20	5.04
6	13.05	12.81	11.47	10.87	9.88	9.73	9.53	10.41	9.49	9.06	8.52	8.15	7.97	6.49	5.75	5.24
7	11.95	12.43	11.28	10.45	9.25	9.07	8.89	10.64	9.55	9.28	7.40	6.94	6.44	6.63	6.01	5.76
8	11.94	12.95	11.03	10.02	9.5	9.26	8.98	10.77	10.27	9.05	7.32	6.95	6.68	7.13	5.94	5.60
9	11.97	13.05	11.21	9.92	9.65	9.36	9.12	10.72	10.44	10.09	7.46	7.06	6.93	7.13	5.77	5.61
10	12.16	13.10	11.23	10.04	9.53	9.36	9.12	10.53	10.31	10.02	7.35	7.03	6.94	6.77	5.84	5.34
11	12.50	13.00	11.09	10.25	9.52	9.24	8.92	10.27	9.76	9.47	7.30	6.83	6.70	7.51	5.99	5.71
12	12.57	12.96	11.14	10.42	9.96	9.59	9.1	10.02	9.68	9.16	7.61	7.16	6.78	7.09	6.44	6.13
13	12.71	12.82	11.11	10.43	10.51	9.71	9.08	10.11	9.80	8.68	7.95	6.71	6.51	7.29	6.03	5.86
14	12.74	12.89	11.12	10.62	10.52	9.63	8.89	10.02	9.74	8.39	7.71	7.01	6.88	5.40	5.01	3.79
15	12.72	13.16	11.02	11.08	10.75	9.84	9	9.75	9.28	8.00	7.78	7.14	6.73	6.67	6.20	4.98
16	12.79	13.12	11.05	11.09	11	10.15	9.3	9.67	9.48	7.97	7.89	7.21	6.89	7.73	6.45	6.01
17	12.85	13.19	11.15	10.98	11.15	10.18	9.37	9.49	9.12	7.71	8.10	7.48	6.92	7.58	7.11	5.85
18	12.43	12.59	11.53	10.92	10.7	10.3	9.21	10.13	9.96	8.65	9.21	8.74	7.95	6.33	5.83	4.88
19	12.97	12.64	11.65	10.88	10.93	10.1	9.21	11.02	10.77	9.30	6.84	6.38	5.62	7.70	6.41	5.94
20	12.40	13.86	11.16	10.82	10.46	9.62	8.91	10.39	10.08	8.94	6.69	6.12	5.24	7.12	5.78	5.57
21	12.21	13.17	11.25	10.63	10.09	9.19	8.54	9.97	9.15	8.45	6.72	6.23	5.27	7.25	5.81	5.67
22	12.35	13.22	11.06	10.58	10.32	9.34	8.77	9.87	9.21	8.38	7.17	6.75	5.52	7.68	6.27	6.11
23	12.17	13.31	11.12	10.54	10.55	9.51	9.01	9.88	9.65	8.34	7.19	6.76	5.84	7.47	6.18	5.92
24	11.80	13.03	11.17	10.48	10.9	10.03	9.37	10.07	9.46	8.54	6.96	6.40	5.83	7.25	5.71	5.34
25	11.59	12.88	11.18	9.93	10.28	9.42	8.87	9.67	9.41	8.36	6.90	6.01	5.49	6.81	4.76	4.41
26	11.41	12.80	11.21	10.27	10.15	9.33	8.77	9.07	8.72	7.68	7.38	6.83	6.00	6.39	5.89	5.06

* The grey cells indicate the thickness of the hardfaced flights below 8 mm (corresponding to probe S, the original flight without deposition).

## Data Availability

The original contributions presented in this study are included in the article. Further inquiries can be directed to the corresponding authors.

## Abbreviations

The following abbreviations are used in this manuscript:

GMAW	Gas Metal Arc Welding
SMAW	Shielded Metal Arc Welding
EAPA	European Asphalt Pavement Association
USD	United States Dollar
DEM	Discrete Element Method
MEK	Methyl-Ethyl-Ketone
PTAW	Plasma Transfer Arc Welding
APS	Asphalt Producers Service
